# Supplementary figures and images for: Morphological peculiarities of the DNA-protein complexes in starved Escherichia coli cells
Source: PLoS One. 2020 Oct 2;15(10):e0231562. doi: 10.1371/journal.pone.0231562 (PMC7531825; doi:10.1371/journal.pone.0231562)

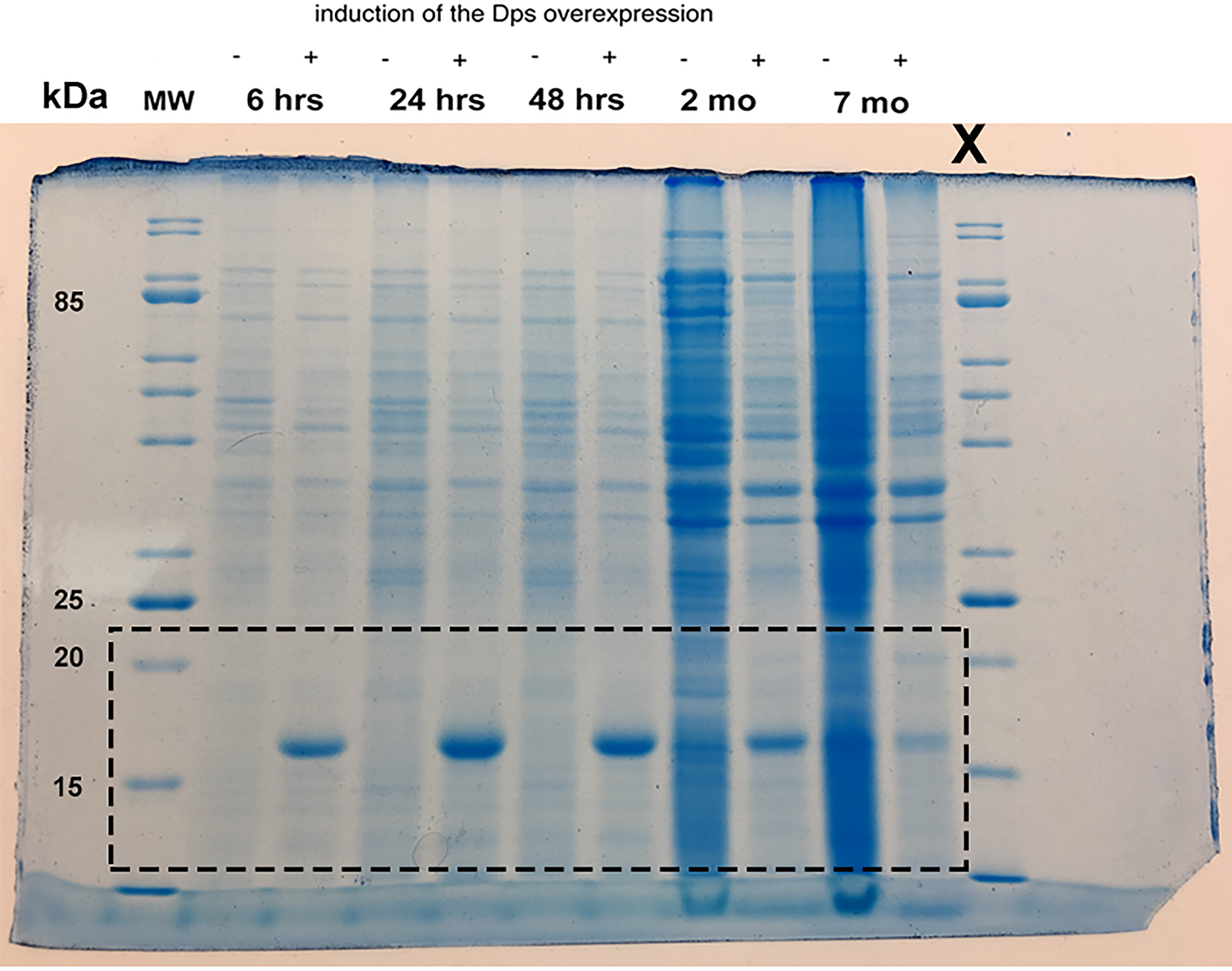

Supplement: S1 Fig — (TIF) [file pone.0231562.s001.tif]

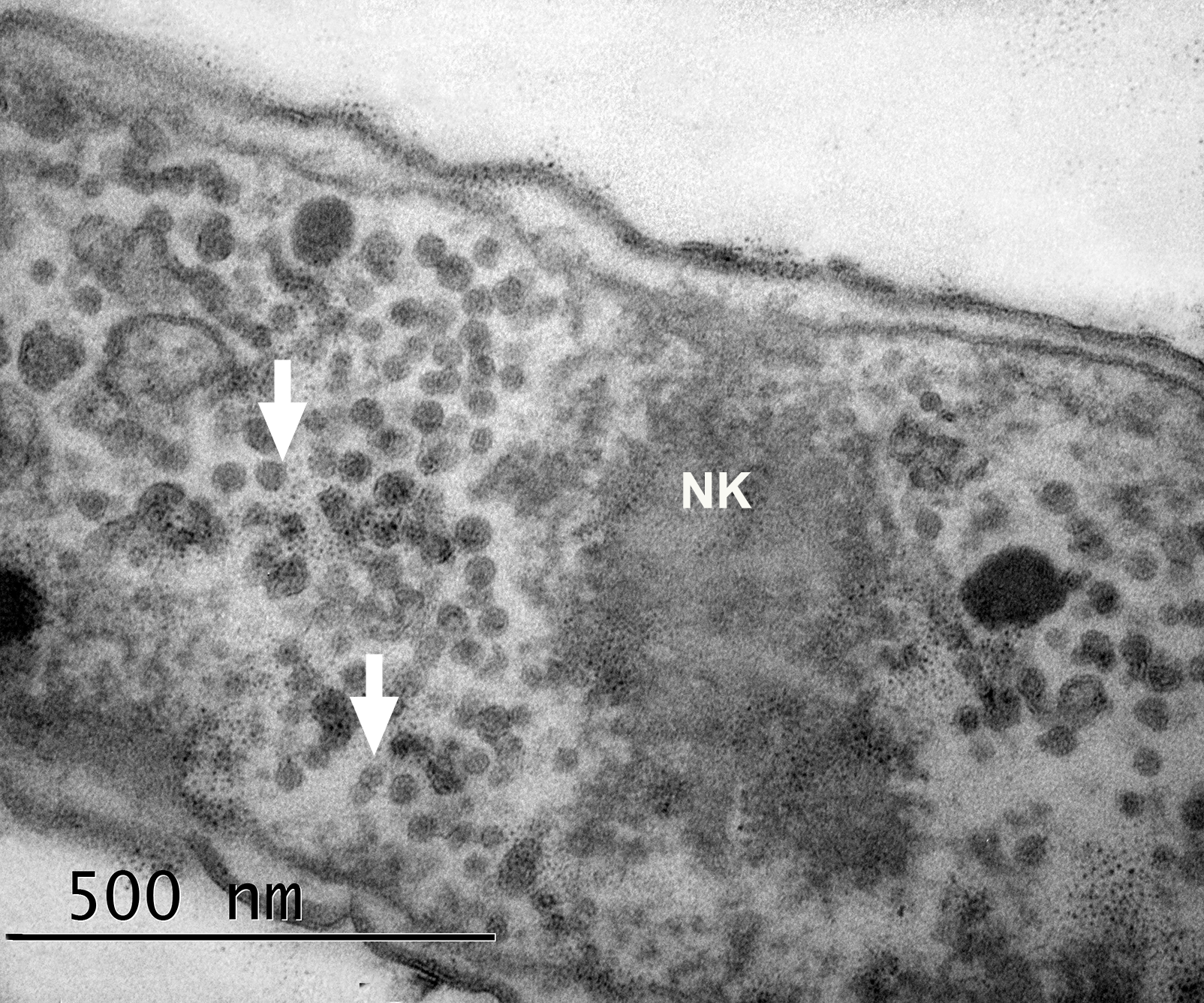

Supplement: S2 Fig — NK–nanocrystalline; arrow—folded nucleosome-like condensate. (TIF) [file pone.0231562.s002.tif]
