# Supplementary material for: Morphological peculiarities of the DNA-protein complexes in starved Escherichia coli cells
Source: PLoS One. 2020 Oct 2;15(10):e0231562. doi: 10.1371/journal.pone.0231562 (PMC7531825; doi:10.1371/journal.pone.0231562)
Supplement: Attachment — Submitted filename: Response to Reviewers.docx [file pone.0231562.s003.docx]

PONE-D-20-08434

We thank the reviewers for their positive and very helpful comments, which we addressed as follows:

*PLOS ONE now requires that authors provide the original uncropped and unadjusted images underlying all blot or gel results reported in a submission’s figures or Supporting Information files.*

In addition to the simplified gel version shown on Fig.1 the original uncropped and unadjusted image is added to Supporting Information files as S1_raw image.

*We note that you have included the phrase “data not shown” in your manuscript. Unfortunately, this does not meet our data sharing requirements. PLOS does not permit references to inaccessible data.*

We added an additional supplemental S2_Figure that demonstrates the co-detection of several types of nano-structures in one cell.

*Reviewer #1: 

The paper is interesting and visually appealing, but there is a number of issues that should be tackled:*

We thank the reviewer for their very encouraging and sympathetic review.

*1. A clarification of the biological nomenclature would be very welcome. I do not understand the precise meaning of *dormant*. Is it that they keep some membrane potential—but can they generate viable colonies? What is *vegetative* in this context (eg Table 1)? How do we know the cells observed under the microscope are alive or dead?*

By vegetative we mean actively growing cells, see the new caption to Table 1.

By dormant we mean a metabolic state of bacteria life entered in response to adverse environmental conditions such as starvation, desiccation, freezing, oxygen deficiency and so on. In the dormant state, all measurable metabolic processes stop, preventing reproduction, development, and repair. When environmental conditions return to being hospitable, the cell will return to its metabolic state of life as it was prior to the dormancy.

In this article, by dormant statе we mean precisely only one meaning of this general term: the state that bacteria take, due to prolonged starvation (see the changed title of the article). We added an explanation on page 11.

Increased synthesis of Dps in *E. coli* cells occurs in the stationary phase, under long starving conditions, allowing to protect the DNA from oxidative stress, heat, acid, alkaline shock, toxic effects, antibiotics, UV radiation, etc. The protective functions of Dps are carried out through the condensation of DNA into several morphologically different types of DNA condensation in dormant (starved) *Escherichia coli* cells, namely: *nanocrystalline, liquid crystalline*, and the *folded nucleosome-like* (see the results of the present article).

Determination of the number of viable (alive) cells was carried out by plating an aliquot from the desired sample onto agar LB medium after a series of required tenfold dilutions. Inoculations were incubated for 3 days at 28°C. Then, the number of grown colonies was counted and, taking into account the dilutions, the number of viable cells in each sample was calculated, expressing them in terms of colony-forming units in ml (CFU/ml).

When working with dormant cells, they were preliminarily revitalized by rinsing in distillate water.

*2. The Achilles's heel of this work is that the interesting structures observed under EM come from cells overexpressing Dps. The wt is mentioned in Table 2, but not analyzed further. It is worrisome that the distribution of the various DNA-protein complexes depends on the overexpression system. BTW, the Table lacks various controls eg strains carrying insert-less plasmids (not just the wt) with and without inducers.*

We thank the reviewer for their useful comments. This issue was discussed in the article. We emphasized that the morphology of the condensed structures depends on the overproduction of the Dps protein. The structures described in the work were found nearly in all the studied strains, both wild-type and mutants. The only exception was the Dps-null mutant, in the dormant form of which only one Dps-DNA structure (*liquid crystalline*) was found. We have included this data in the article (Table 2) and discussed it in more detail in the results section.

Wild strains without plasmids were served as a control in the present work. We have inserted additional data on another control wild strain K12MG1655 into the article in response to the reviewer’s comment. It became clear (see Table 2) that the distribution of the condensed structures in this strain is close to that in wild strain Top 10.

We believe that experiments with two control wild strains in some sense replace the experiment with an insert-less plasmid, since the insert-less plasmid did not carry any information about other proteins related to DNA stress protection.

*3. Is Dps protein essential? It would be highly informative to inspect starved cells lacking the protein. The nucleoid has many other associated proteins which could contribute to the structures shown in the figures.*

We thank the reviewer for their useful suggestion and performed the experiment on cells with Dps knockout. We added a paragraph to the methods section describing the production of the Dps-null mutant on page 7. The description of the condensed structures in the K12 Dps-null strain are shown in Table 2.

The morphology of liquid crystalline assembly in dormant *E. coli* cells K12 Dps-null strain is shown on this Figure.

Reviewer #2: 

*1.Title: please try to shorten it.*

We shortened the title, according to the reviewer’s suggestion.

The new title is: Morphological peculiarities of the DNA-protein complexes in starved Escherichia coli cells

*2. The experimental procedures used are appropriate to ask the proposed questions. I encourage the improvement of the statistical analysis of the observations summarized in Table 2, including some statistical parameters (i.e., the standard deviation of the mean values, or equivalent) to allow the reader to evaluate if the abundance of the difference morphologies found is significant within experimental uncertainty or not.*

Standard deviations of the mean values were estimated on the basis of the unbiased dispersion estimate and are shown in a new version of Table 2 of the article

*3. The manuscript will improve if the authors further elaborate on the significance of the novel structures found here (namely the ones denoted as folded nucleosome-like structures). In this regard, Figure 6 would need further improvements. How these results and the interpretations of them agree (or contrast) with previously published work?*

Figure 6 has been changed and improved. The comparison between eukaryotic and prokaryotic DNA compactization is shown in the schematic on Fig. 6. We consider that it contains some additional new information on the prokaryote DNA compactization in the dormant cell state.

*4. In the conclusions, could the authors define more precisely the unsettled points for future explorations?*

We thank the reviewer for the suggestion. Many questions remain to be addressed in future research. The dormant metabolic state of the bacteria studied in this article was entered as a response to adverse environmental conditions, such as prolonged starvation. There are many other ways to obtain dormant states as a response to other adverse environmental conditions, such as extreme desiccation, freezing, oxygen deficiency, factors of suspended animation (anabiosis), etc. Such dormant forms differ from each other in their properties and their ability to survive, which, apparently, should be associated with the difference in their ultrastructure. In further studies, we propose to study the results of DNA condensation in dormant cells obtained in the different ways mentioned. This will help acquire a more complete understanding of the ability of bacterial cells to form various ordered condensed DNA structures with nucleoid associated proteins (NAP, including Dps) and to understand why cells need heterogeneity for survival. We consider that such type of research is promising, since it may bring some understanding of the mechanisms of resistance of bacteria and will allow to solve many problems of bioengineering and medicine. Let us recall that the universal adaptive response of microorganisms to starvation is of specific interest, because it often coincides with the resistance of pathogenic bacteria to antibiotics; this represents one of the most important medical problems in the world to date.

A new paragraph has been added in the Discussion section on page 23.

*5. What are the principles, relationships, and generalizations shown by the results?*

Our results show that condensed DNA structures are drastically different in actively growing and dormant states of *Escherichia coli* cells. In the present paper, we have observed three types of DNA condensation in dormant *Escherichia coli cells*. First two: *nanocrystalline* and *liquid crystalline* structures, are typical for inanimate nature. The third one: *folded nucleosome-like*, is novel for an inanimate nature structure and may be the result of complex interaction and multiple folding of long DNA molecules around the Dps protein and its associates. Multiple types of DNA condensation in the same dormant *E. coli* cell increase the chances of the cells for survival and for rapid resumption of growth when conditions turn out to be favorable.

We have added a description to page 22.
